# Bilateral Valgus Slipped Capital Femoral Epiphysis in an 11-year-old Girl

**DOI:** 10.7759/cureus.3598

**Published:** 2018-11-15

**Authors:** Sulaiman F Almedaifer, Abdullah J. AlShehri, Thamer S Alhussainan

**Affiliations:** 1 Orthopedic Surgery, King Faisal Specialist Hospital and Research Centre, Riyadh, SAU

**Keywords:** slipped capital femoral epiphysis, valgus, bilateral, klein’s line

## Abstract

Valgus slipped capital femoral epiphysis (SCFE) is very infrequent, and it is characterized by a superolateral displacement of the epiphysis on the metaphysis. To date, less than 100 cases of valgus SCFE have been described in the literature. Bilaterality of valgus SCFE is extremely rare, and it presents management challenges to the treating orthopedic surgeons. Herein, we report the case of an 11-year-old Saudi Arabian girl presented to clinic with a one-year history of bilateral hip pain and limping. Past medical history was negative for endocrinopathies, hemoglobinopathies, bone disorders, trauma or radiation therapy to the pelvis. On physical examination, the patient looked tall and obese. On clinical examination, the patient showed a waddling gait and an external rotation on walking. A frog-leg lateral radiograph showed bilateral SCFE with a valgus deformity. The right and left femoral neck-shaft angles measured 154.3 and 148.2 degrees, respectively. Computed tomography (CT) scan suggested a moderate bilateral posterior slippage of femoral heads; the right and left femoral head-neck angles measured 60 and 52 degrees, respectively. A final diagnosis of bilateral valgus SCFE was established. Consequently, the patient underwent bilateral percutaneous in situ pinning with single cannulated screws. Postoperatively, the patient made an uneventful recovery. At one-year follow-up, hip radiograph showed bilateral atypical narrowing of the joint space and suspected chondrolysis and the physis of both proximal femoral heads were fused. On the right side, the fixating screw was penetrating into the articular surface of the femoral head with some osteoarthritic changes. Considering the patient’s worsening situation, it was decided to perform a revisional surgery. The revisional surgery included the removal of bilateral screws and administration of local steroids and analgesics for pain control. Post-revisional surgery at three months, though the patient was limping with a pelvic tilt, she was able to ambulate with the aid of axillary crutches.

## Introduction

Slipped capital femoral epiphysis (SCFE) is a common hip disorder of adolescence [1]. There are two forms of SCFE, namely varus and valgus SCFE. Varus SCFE is the most frequently encountered form, and it is characterized by a posteromedial displacement of the epiphysis on the metaphysis. Conversely, valgus SCFE is very infrequent, and it is characterized by a superolateral displacement of the epiphysis on the metaphysis [1]. The first ever case of valgus SCFE was reported in 1926 by Müller [1-2]. To date, less than 100 cases of valgus SCFE have been described in the literature [2]. The vast majority of valgus SCFE cases are unilateral. On the other hand, cases of bilateral valgus SCFE are extremely rare and present management-related challenges to the treating orthopedic surgeons. To the best of our knowledge, less than 25 cases of bilateral valgus SCFE have been reported so far in the English literature. Herein, we report the case of bilateral valgus SCFE in an 11-year-old Saudi girl who presented to the clinic with a one-year history of bilateral hip pain and limping.

## Case presentation

An 11-year-old Saudi Arabian girl presented to the clinic with a one-year history of bilateral hip pain and limping. The symptoms progressed over two weeks. Past medical history was negative for endocrinopathies, hemoglobinopathies, bone disorders, trauma or radiation therapy to the pelvis. She was delivered by spontaneous vaginal delivery at term with no neonatal intensive care unit (ICU) admission.

Laboratory tests were normal for serum insulin-like growth factor-1 (IGF-1), thyroid-stimulating hormone (TSH), follicle-stimulating hormone (FSH), luteinizing hormone (LH), adrenocorticotropic hormone and prolactin.

On general physical examination, the patient looked tall and obese and ambulating with axillary crutches. The patient’s height and weight were at the 90th percentile according to the Centers for Disease Control and Prevention (CDC) growth charts.

On clinical examination, the patient showed a waddling gait and an external rotation on walking. Both hips displayed obligatory external rotation (+10 degrees) with limited hip flexion and internal rotation (-10 degrees). Furthermore, both hips exhibited limited abduction and extension (-10 degrees). Leg-length discrepancies were observed; the right leg was adducted, whereas the left leg was externally rotated. The patient was ambulating with the assistance of axillary crutches. The right leg was noted to be in a slightly worse condition than the left leg on the account that it revealed more external rotation while walking.

**Figure 1:**
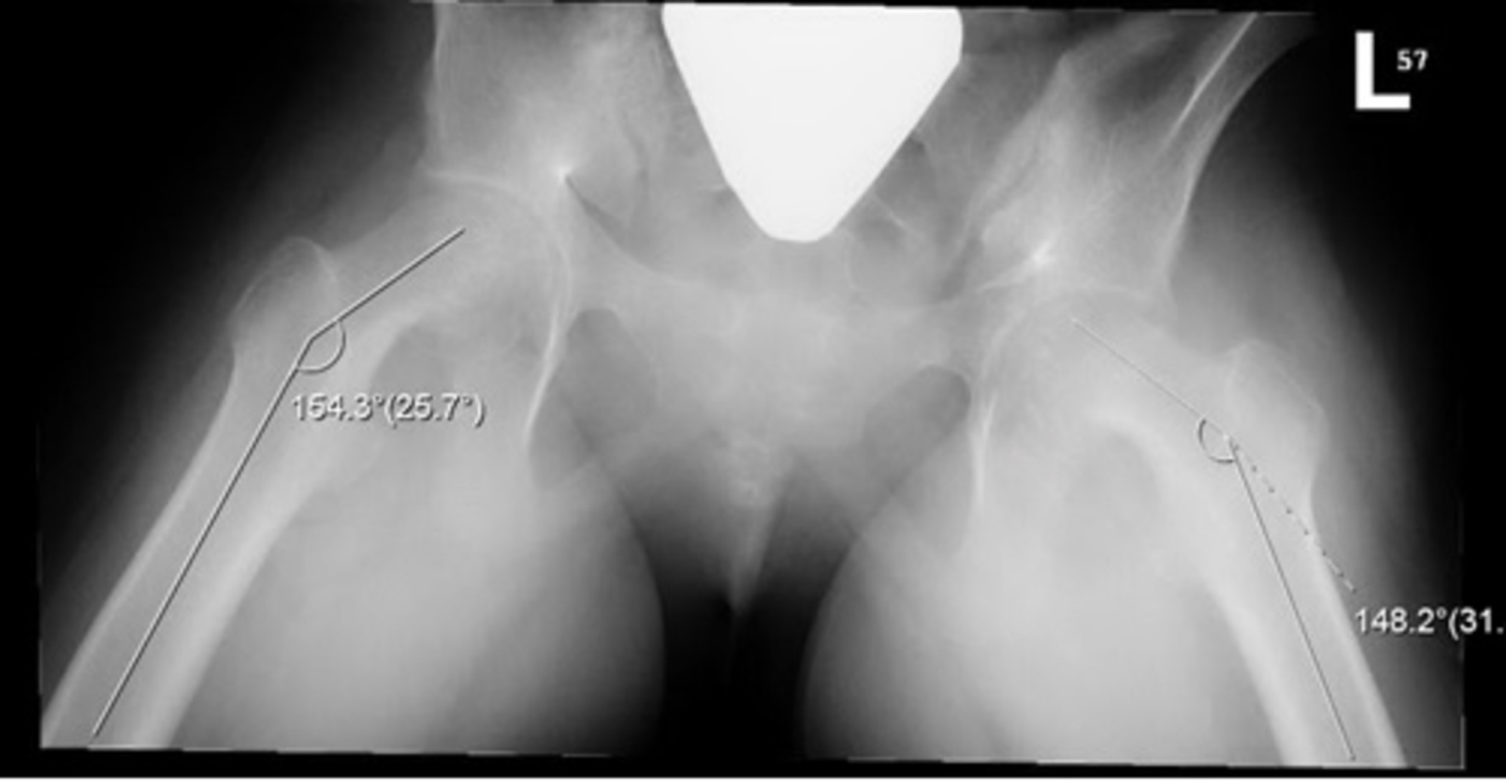
Frog-leg lateral radiograph The radiograph showed bilateral SCFE with valgus deformity. The right and left femoral neck-shaft angles measured 154.3 and 148.2 degrees, respectively. SCFE: slipped capital femoral epiphysis

On anteroposterior (AP) X-ray view, the Klein’s line was normal bilaterally. A frog-leg lateral radiograph (X-ray) showed bilateral SCFE with valgus deformity (Figure 1). The right and left femoral neck-shaft angles measured 154.3 and 148.2 degrees, respectively. A generalized osteopenia of the hip joints was observed. There was no avascular necrosis, dislocation or subluxation.

**Figure 2:**
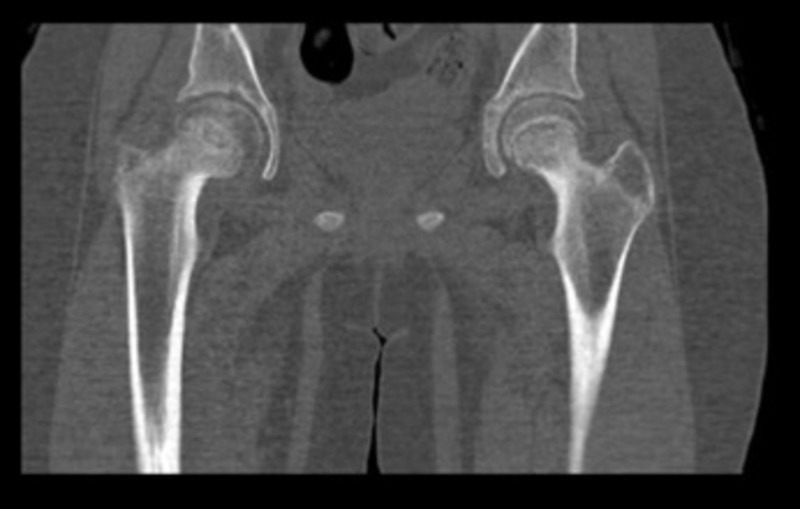
Coronal CT scan The CT scan showed a moderate bilateral posterior slippage of femoral heads; the right and left femoral head-neck angles measured 60 and 52 degrees, respectively. CT: computed tomography

Preoperative computed tomography (CT) scan suggested a moderate bilateral posterior slippage of femoral heads; the right and left femoral head-neck angles measured 60 and 52 degrees, respectively (Figure 2).

**Figure 3:**
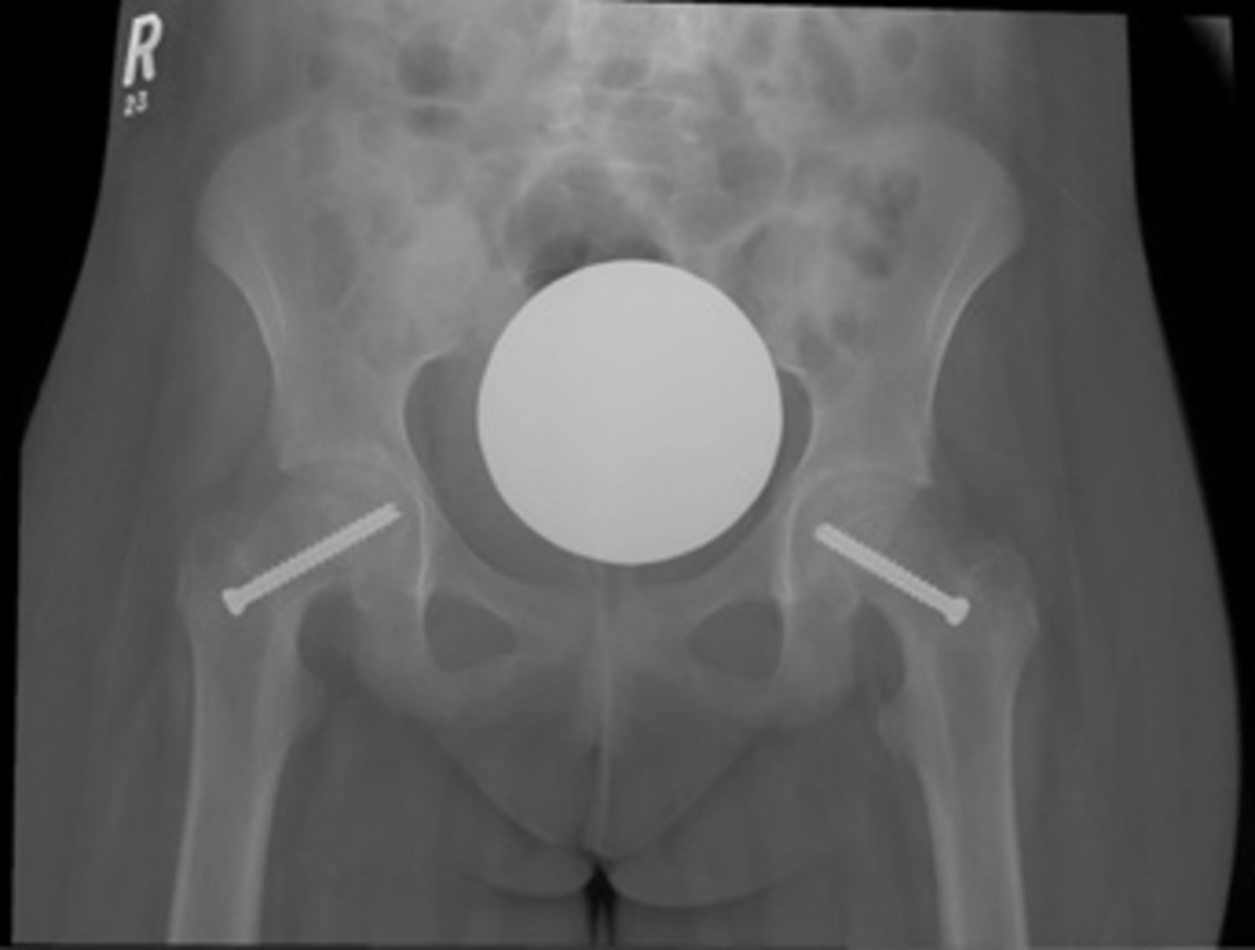
Intraoperative fluoroscopy The intraoperative fluoroscopic image showed bilateral percutaneous in situ pinning with single cannulated screws.

A final diagnosis of bilateral valgus SCFE was established. Consequently, the patient underwent bilateral percutaneous in-situ pinning with size 50-mm and length 6.5-mm single cannulated screws (Figure 3). The screws were placed under fluoroscopy guidance. Screws placement was satisfactory with no sign of acetabular impingement in all fluoroscopic planes.

Postoperatively, the patient made an uneventful recovery. She was discharged on a wheelchair for mobilization and instructed to avoid weight-bearing activities for the first three months.

**Figure 4:**
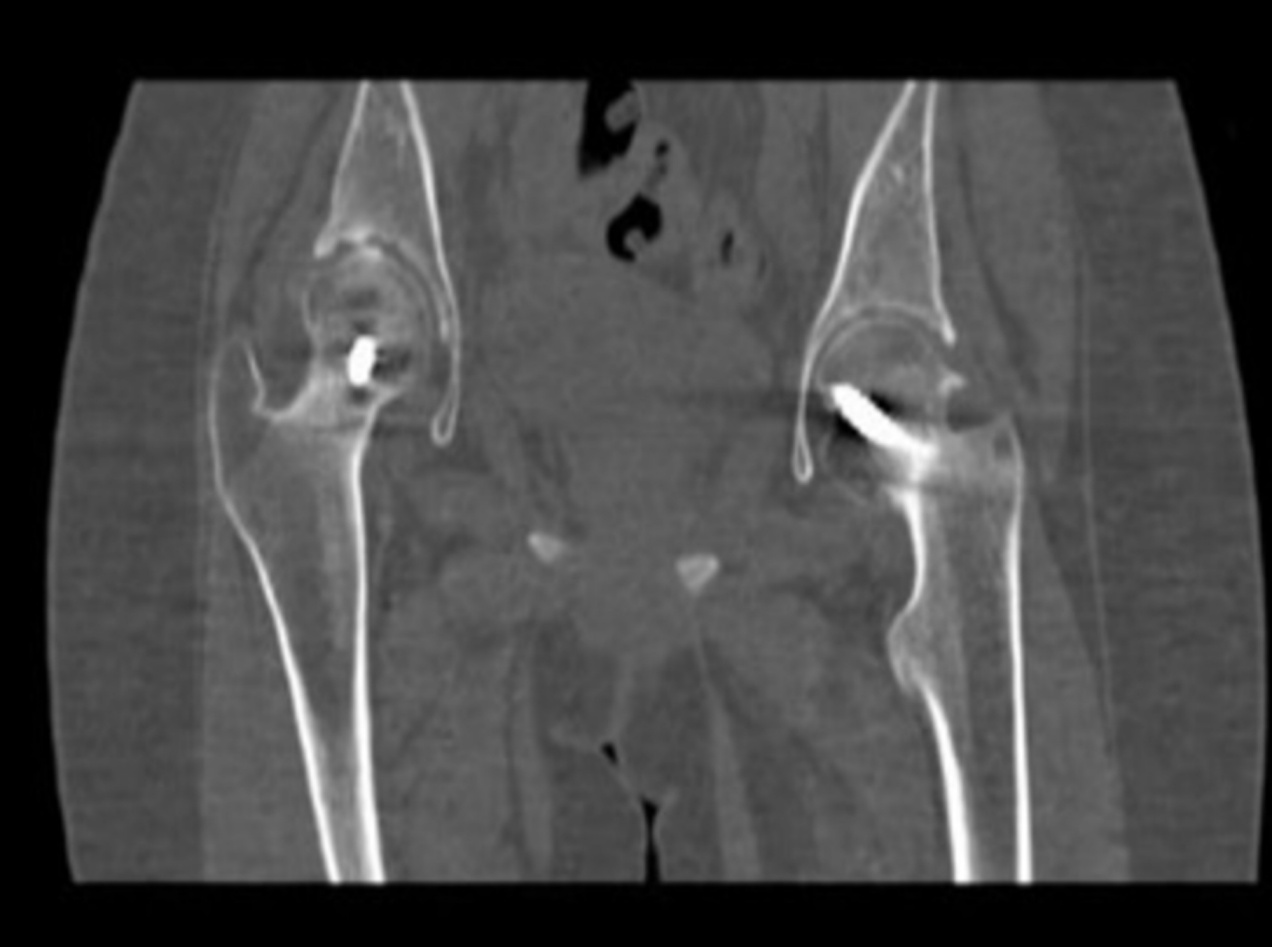
One-year postoperative coronal CT scan The left hip showed subchondral sclerosis with lateral osteophytes. On the right side, the fixating screw was noted to penetrate into the articular surface of the femoral head with some osteoarthritic changes. CT: computed tomography CT: computed tomography

At one-year follow-up, hip radiograph showed bilateral atypical narrowing of the joint space and suspected chondrolysis. In addition, the physes of both proximal femoral heads were fused. CT report revealed left hip subchondral sclerosis with lateral osteophytes (Figure 4). On the right side, the fixating screw was noted to be penetrating into the articular surface of the femoral head with some osteoarthritic changes. After a thorough discussion of the patient’s worsening situation, it was decided to perform a revisional surgery. The revisional surgery included the removal of bilateral screws, as well as administration of local steroids and analgesics for pain control. Post-revisional surgery at three months, though the patient was limping with a pelvic tilt, she was able to ambulate with the aid of axillary crutches. 

## Discussion

Our patient presented with a one-year history of bilateral hip pain and limping. Generally, hip pain is the most frequent presenting symptom in patients with SCFE. However, around 15% of patients may present with an unusual isolated knee or thigh pain, which may delay early diagnosis, and eventually lead to deteriorating prognosis [3].

Our patient was diagnosed with bilateral valgus SCFE. To date, less than 100 cases of valgus SCFE have been described in the literature [2]. In 2006, Loder and colleagues reviewed the literature on varus and valgus SCFE [1]; the approximated frequency of valgus SCFE was less than 2%. In 2010, a single-institutional experience from the United States of America reported a valgus SCFE prevalence of 4.7% (12 of 258 patients) [4]. On the other hand, in 2013, a single-institutional experience from Poland reported a valgus SCFE prevalence of 9.6% (11 of 115 patients) [5]. When compared to classical varus SCFE, valgus SCFE had a greater predilection for females [1-5], and this finding matched to our patient’s gender in the case.

Obesity [6], endocrine disorders (for instance, hypothyroidism and growth hormone deficiency) [7] and exposure to radiation therapy [8] are well-established risk factors for the development of SCFE. More specifically, an association between the endocrine disorders (particularly panhypopituitarism) and valgus SCFE has been well-demonstrated [4,9]. The only risk factor identified in our patient was teenager obesity based on CDC’s BMI-for-age percentile growth charts.

Proper interpretation of radiographs is very critical in minimizing the chances of missing the diagnosis of SCFE [10]. Furthermore, early diagnosis of SCFE is crucial in preventing the long-term crippling consequences. In a normal AP radiograph, Klein's line refers to a line drawn along the superior femoral neck that interests the lateral portion of the femoral neck. Klein's line has been traditionally used to diagnose SCFE on AP view [11]. Although Klein’s line is a supportive tool in diagnosing SCFE, it has been criticized for lack of sensitivity and failure to detect SCFE [12-13]. A modification of the Klein’s line has been suggested in an attempt to increase the sensitivity of diagnosing SCFE in the AP radiograph [12]. The modified Klein’s line takes into consideration the epiphyseal width lateral to the Klein’s line; a difference of 2 mm or more between hips greatly suggests SCFE with a higher sensitivity [12]. However, in valgus SCFE, the Klein’s line will almost always be normal, and this has underscored the necessity for lateral radiographs to be completed in all children with hip pain [1]. Relative to our case, the Klein’s line was normal on an AP radiograph, and a lateral (frog-leg) radiograph identified valgus SCFE. Lateral radiographs (frog-leg views or cross-table lateral views) should be always performed in assessing patients for SCFE [13], particularly valgus SCFE.

In our case, the right and left femoral neck-shaft angles were 154.3 and 148.2 degrees, respectively. Moreover, the right and left femoral head-neck angles measured 60 and 52 degrees, respectively. All these findings were greater than the normal ranges and thus were in favor of the diagnosis of bilateral valgus SCFE. Increased neck-shaft angle, increased femoral anteversion, horizontal physis and coxa valga have been suggested as critical contributors to the pathogenesis of valgus SCFE [1,4,9,14].

Differential diagnoses in our case comprised femoral neck fracture and hip dysplasia. However, these differential diagnoses were excluded in consideration of the patient’s age, history and lack of supporting clinical signs. Correct diagnosis and prompt management of SCFE is critical to prevent complications, such as avascular necrosis of the hip, femoro-acetabular impingement, chondrolysis, osteoarthritis and permanent bed-ridden disability [15].  

To the best of our knowledge, less than 25 cases of bilateral valgus SCFE have been reported in the literature [1,4,16-17]. Furthermore, we report the first ever case of bilateral valgus SCFE from Saudi Arabia. Such cases present management challenges to the treating orthopedic surgeons. Management generally encompasses in situ pinning and inter- or sub-trochanteric osteotomy [14]. In valgus SCFE, the posterolateral displacement of the femoral epiphysis makes in situ pinning more challenging. This is because the entry point for the screw has to be positioned more medially, which in turn endangers the underlying femoral neurovascular bundle [1,4,14,17]. Therefore, it is highly advised to perform a mini-longitudinal incision to guard the neurovascular bundle during screw fixation. Owing to the juxtaposition of the neurovascular bundle and the associated danger of a medial approach, several authors recommend an open surgery (osteotomy) approach to protect the neurovascular structures [1,14]. The potential complications of the osteotomy (for example, hematoma, fracture, osteonecrosis and cutaneous nerve dysesthesia) should be contemplated [18]. As of now, there is no optimal approach to the management of bilateral valgus SCFE. Until evidence-based consensus becomes available, management should be guided by a clinical judgment that takes into consideration several issues, such as the surgeon’s experience, patient’s preference and weighing the risks versus benefits. In our case, the patient was managed with bilateral percutaneous in-situ pinning, which was complicated postoperatively, and a revisional surgery was instituted later.

## Conclusions

Valgus SCFE is very infrequent, and it is characterized by a superolateral displacement of the epiphysis on the metaphysis. Bilaterality is extremely uncommon and presents management challenges to the treating orthopedic surgeons. There is no optimal approach for management of bilateral valgus SCFE. In valgus SCFE, the posterolateral displacement of the femoral epiphysis makes in situ pinning more challenging. This is because the entry point for the screw has to be positioned more medially, which in turn endangers the underlying femoral neurovascular bundle. In valgus SCFE, the Klein’s line will almost always be normal, and therefore lateral radiographs should be completed in all children with hip pain.
